# The Opioid System in Temporal Lobe Epilepsy: Functional Role and Therapeutic Potential

**DOI:** 10.3389/fnmol.2017.00245

**Published:** 2017-08-07

**Authors:** Johannes Burtscher, Christoph Schwarzer

**Affiliations:** Department of Pharmacology, Medical University of Innsbruck Innsbruck, Austria

**Keywords:** hippocampus, dynorphin, enkephalin, endorphin, seizures, kappa-opioid receptor, mu-opioid receptor, delta-opioid receptor

## Abstract

Temporal lobe epilepsy is considered to be one of the most common and severe forms of focal epilepsies. Patients often develop cognitive deficits and emotional blunting along the progression of the disease. The high incidence of resistance to antiepileptic drugs and a frequent lack of admissibility to surgery poses an unmet medical challenge. In the urgent quest of novel treatment strategies, neuropeptides are interesting candidates, however, their therapeutic potential has not yet been exploited. This review focuses on the functional role of the endogenous opioid system with respect to temporal lobe epilepsy, specifically in the hippocampus. The role of dynorphins and kappa opioid receptors (KOPr) as modulators of neuronal excitability is well understood: both the reduced release of glutamate as well of postsynaptic hyperpolarization were shown in glutamatergic neurons. In line with this, low levels of dynorphin in humans and mice increase the risk of epilepsy development. The role of enkephalins is not understood so well. On one hand, some agonists of the delta opioid receptors (DOPr) display pro-convulsant properties probably through inhibition of GABAergic interneurons. On the other hand, enkephalins play a neuro-protective role under hypoxic or anoxic conditions, most probably through positive effects on mitochondrial function. Despite the supposed absence of endorphins in the hippocampus, exogenous activation of the mu opioid receptors (MOPr) induces pro-convulsant effects. Recently-expanded knowledge of the complex ways opioid receptors ligands elicit their effects (including biased agonism, mixed binding, and opioid receptor heteromers), opens up exciting new therapeutic potentials with regards to seizures and epilepsy. Potential adverse side effects of KOPr agonists may be minimized through functional selectivity. Preclinical data suggest a high potential of such compounds to control seizures, with a strong predictive validity toward human patients. The discovery of DOPr-agonists without proconvulsant potential stimulates the research on the therapeutic use of neuroprotective potential of the enkephalin/DOPr system.

## Introduction

With a prevalence of 1–2% worldwide, epilepsy is one of the most frequent neurological diseases affecting people of all ages (Thurman et al., 2011; WHO, 2015). Of the 870 million people living in the European Region, over 5 million suffer from epilepsy. Epilepsy has major adverse effects on both social and psychological well-being, including social isolation, stigmatization, or disability, thus, resulting in lower educational achievement and worse employment outcomes (WHO, 2015). In line with this, the International League Against Epilepsy (ILAE) defined epilepsy as “a disorder of the brain characterized by an enduring predisposition to generate epileptic seizures and by the neurobiologic, cognitive, psychological, and social consequences of this condition.”

The term *epilepsy* comprises of a group of chronic neurological diseases that can be characterized by epileptic seizures, as a result of excessive electrical discharges in a group of brain cells (Fisher et al., 2005, 2014). *Epileptic seizures* are episodes that vary from brief and nearly undetectable to prolonged convulsions, and may involve a part of the brain (partial) or multiple brain centers or the entire brain (generalized), and are sometimes accompanied with a loss of consciousness and control of bowel or bladder functions. The cause of epilepsy is mostly unknown (*ca*. 60%), although several patients have a history of brain injury, stroke, brain tumor, and substance use disorders (Berkovic et al., 2006; Thurman et al., 2011; WHO, 2015). Genetic, congenital, or developmental epilepsies are more common among younger people, while brain tumors and stroke are more common causes in older people.

About 70% of all epilepsy patients suffer from focal seizures arising from a distinct brain region, the temporal lobe. Mesial temporal lobe epilepsy (mTLE, with the hippocampus as epileptogenic focus) is considered as one of the most frequent types of epilepsy (Blumcke et al., 2012; Goldberg and Coulter, 2013). mTLE with hippocampal sclerosis represents one of the most refractory forms of human epilepsy (Asadi-Pooya et al., 2017). One main factor responsible for neuronal losses and seizure induction is excessive glutamate release (Meldrum, 2002), which may result from impaired inhibitory signaling. The mainstay treatment of epilepsy relies on *antiepileptic drugs (AEDs)*, mostly for the person's entire life. Notably, 30–50% of the patients are refractory to the presently available pharmacological treatments (Laxer et al., 2014). Moreover, the current pharmacotherapies of epilepsy causes a number of side effects (e.g., sedation, nausea, depression, headache, ataxia) in 10 to 90% of people (Eadie, 2012; Perucca and Gilliam, 2012). In 2008, the FDA issued a black-box warning that several AEDs increased the risk of suicidal thoughts and behavior among the users (Mula and Sander, 2013). In patients whose seizures cannot be efficiently controlled by AEDs or neuro-stimulation, surgical resection of the epileptogenic focus remains the ultimate solution (Duncan, 2007; Bergey, 2013). Besides, only about 50–80% reach seizure freedom for at least 1 year (Spencer and Huh, 2008).

In the quest for alternative treatment options, neuropeptides have received an increasing attention. Neuropeptide systems have been demonstrated to play crucial roles in the modulation of neuronal excitability. Several neuropeptides, such as neuropeptide Y, galanin, somatostatin, ghrelin, and dynorphin, have been reported to have direct antiepileptic and antiepileptogenic effects, and they represent promising potential drug targets (Kovac and Walker, 2013). The aim of this review is to reflect upon the opioid system's function in epileptogenesis and temporal lobe epilepsy as well as their therapeutic potentials.

## The endogenous opioid system in the hippocampus: general aspects

The three classical opioid receptors, the kappa opioid receptor (KOPr), delta opioid receptor (DOPr), and mu opioid receptor (MOPr), are Gi/o-coupled 7-transmembrane domain proteins, and they share highly homologous protein sequences (60% amino acid sequence identity) including a common opioid receptor binding pocket within the helical transmembrane core. The extracellular domains are crucial for selectivity, however, the opioid system is fairly promiscuous and the affinities of dynorphins, endorphins, and enkephalins to KOPr, MOPr, and DOPr vary only in the range of one potency (Schwarzer, 2009).

The opioid receptor-like (ORL)-1 receptor was also classified as an opioid receptor due to its genetic sequence homology to other opioid receptors. It displays, however, distinct pharmacological properties, and therefore, is not considered to be a classical opioid receptor. Although ORL-1 and its endogenous ligand nociceptin might be involved in seizures and epilepsy (Bregola et al., 2002a,b; Binaschi et al., 2003; Aparicio et al., 2004; Rocha et al., 2007, 2009), it will not be discussed in this review.

### The dynorphin/KOPr system

Although there is only one prodynorphin (pDyn) gene known in mammals, different splice variants (Horikawa et al., 1983; Telkov et al., 1998; Nikoshkov et al., 2005), and a variety of different mature peptides, were reported from brain (Yakovleva et al., 2006). *In vitro* studies of different pDyn-derived peptides applied to KOPr suggested a rank order of potency with Dyn A1-17 > (10–20 times) BigDyn = Dyn B = Dyn B 1-29 = α-neo-endorphin > (10–20 times) Dyn A 1-8 = β-neo-endorphin (James et al., 1984). Regional regulation of the trafficking and processing of pDyn at synapses may be important for the fine-tuning of synaptic transmission (Yakovleva et al., 2006).

High pDyn mRNA expression was observed in the amygdala, entorhinal cortex, dentate gyrus, nucleus accumbens, dorsomedial hypothalamus, and premammillary nucleus in humans (Hurd, 1996; Nikoshkov et al., 2005) and rodents (Morris et al., 1986; Merchenthaler et al., 1997; Lin et al., 2006). In the limbic system, strong expression of pDyn mRNA was noted in human (Hurd, 1996), while entorhinal cortex lacks expression in rat (Merchenthaler et al., 1997) or mouse (Lin et al., 2006). Neurons of the central amygdala contain the highest amounts of pDyn in rodents, in human brain, cortical subnuclei express higher amounts of pDyn (Capper-Loup and Kaelin-Lang, 2008).

Immunoreactivy and mRNA distribution display little mismatch (Khachaturian et al., 1982; Vincent et al., 1982b; Fallon and Leslie, 1986). Immunoreactive fibers, such as hippocampal mossy fibers or local circuits in the cortex and amygdala were found (Vincent et al., 1982a; Weber and Barchas, 1983; Code and Fallon, 1986; Fallon and Leslie, 1986). Ultrastructural evidence suggests the presence of Dyn also in dendrites (Van Bockstaele et al., 1995; Hara et al., 2006). In fact, beside the N-type calcium channel-mediated Dyn release at axon terminals, L-type channel-dependent somatodendritic Dyn release was proposed to play an important functional role (Simmons et al., 1995). The distribution of KOPr suggests axonal and dendritic auto- and/or heteroreceptors. How this complex situation may influence neuronal excitability is best demonstrated in the hippocampal granule cells. KOPr on the dendrites of hippocampal granule cells (Mathieu-Kia et al., 2001) may be activated by the Dyn originating from perforant path fiber in humans (Hurd, 1996), or from granule cell dendrites in guinea pigs (Simmons et al., 1995). This is supported by potentially presynaptic KOPr mediated inhibition of perforant path terminals upon the stimulation of hippocampal granule cells (Wagner et al., 1993; Drake et al., 1994). Besides guinea pig, other rodents too display such effects, closely depending on the presence of kappa opioid receptors on the perforant path terminals (Salin et al., 1995). The KOPr present in axo-axonal synapses of mossy fibers mediate heterosynaptic inhibition of neighboring mossy fibers (Weisskopf et al., 1993). CA3 pyramidal neurons may by hyperpolarized through postsynaptic KOPr. Dyn acting on KOPr placed on CA1 pyramidal cells may be derived from perforant path fibers in humans. Functionally, KOPr were shown to be involved in the modulation of hippocampal transmission and LTP (Wagner et al., 1993; Salin et al., 1995; Terman et al., 2000; Huge et al., 2009). Beside glutamatergic neurons, some groups of GABAergic interneurons also express KOPr (Racz and Halasy, 2002). The potential inhibition of inhibitory neurons suggests some excitatory effects under certain conditions.

Taken together, the Dyn/KOPr system is ideally positioned to modulate synaptic transmission at all excitatory synapses of the hippocampus. Most prominent is the control of the granule cells, which are considered a main input filter of the limbic system.

### The enkephalin/DOPr system

Met- and Leu-enkephalin (Enk) are pentapetides encoded in the proenkephalin gene. Moreover, Met-Enk can be processed from proopiomelanocortin (POMC) and Leu-Enk from pDyn. The strongest expression of Enk is found in the basal ganglia, with comparably low expression levels in the hippocampus (Miller and Pickel, 1980). In the rat hippocampus, Enk immunoreactivity was observed in the lateral perforant path and a small number of morphologically characteristic granule cells (Gall et al., 1981; Stengaardpedersen, 1983). Immunoreactivity was also observed in mossy fibers and pyramidal cells in the area CA4 of rats (Stengaardpedersen, 1983), however at a very low level. Low levels of immunoreactivity in rodents are in line with low levels of Met-Enk mRNA (Bing et al., 1997; Schwarzer and Sperk, 1998). In human hippocampi, Enk immunoreactivity was observed in numerous granule cells, interneurons in the molecular layer, as well as pyramidal cells in the hippocampus proper and subiculum (Kulmala, 1985). However, no Enk was observed in the primate perforant path fibers (Gall, 1988). Species differences in Enk immunoreactivity in the hippocampus proper appear to depend on the expression of Enk in the perforant path.

Enks preferentially bind to DOPr, but with only 10-fold lower affinity to MOPr (Clarke et al., 2003). Also, DOPr has a relatively high affinity for β-endorphin (Hughes et al., 1975). The action of DOPr and also that of MOPr in the hippocampus is mainly disinhibitory (Zieglgansberger et al., 1979): MOPr and DOPr activation reduce GABAergic input, thus causing disinhibition (Neumaier et al., 1988) and thereby facilitating synaptic plasticity and seizure susceptibility (Cohen et al., 1992; Morris and Johnston, 1995). The location of Enk in the hippocampus supports the notion of the Enk/DOPr's role in the modulation of inhibitory transmission; Leu-Enk-immunoreactive terminals are often close to GABAergic terminals, perikarya, and dendrites (Commons and Milner, 1996). In mouse hippocampi, DOPr are mainly located presynaptically on inhibitory GABAergic interneurons with some intracellularly located receptors in the pyramidal and granule cells (Rezai et al., 2012). Whether these intracellular receptors represent a pool of spare receptors or serve specific function is unclear. Activation of DOPr in the hippocampus inhibits spontaneous GABA release (Lupica, 1995) and results in net excitatory potential (Drake et al., 2007). Generally, DOPr activation inhibits intracellular cAMP formation and exerts modulatory effects on Ca^2+^ and K^+^ channels and other such 2nd messenger actions (Quock et al., 1999).

It is important to consider that the Enk/DOPr system is very dynamic; owing to agonist-induced internalization and “cross-talk” with other neurotransmitter-systems. Thus, there are several lines of evidence suggesting MOPr/DOPr crosstalk and heterodimerization (for review see Peppin and Raffa, 2015). Thus, MOPr-dependent effects on migration of intracellularly localized DOPr to the membrane surface (Cahill et al., 2003; Morinville et al., 2004) were described through inflammation. Dynamic receptor synthesis and degradation (reviewed by van Rijn et al., 2013), as well as the fast turnover rates of Enk (Hughes et al., 1975; Simantov and Snyder, 1976) are important components of this flexibility.

Due to the location of Enk/DOPr it's regulatory role is considered mostly on modulation of inhibitory signaling. Inhibition of GABAergic interneurons on one hand facilitates excitatory signaling, on the other hand may loosen synchronization and control.

### The endorphin/MOPr system

α-, β-, γ- and δ-endorphins are processed from proopiomelanocortin (POMC), which is expressed by interneurons of the dentate gyrus (Niikura et al., 2013). β-endorphin has been reported to be present in the hippocampus (Zakarian and Smyth, 1979, 1982), but these findings were not reproduced in later reports (Chavkin et al., 1985; Drake et al., 2007). α- and β-neoendorphin can be processed from pDyn, which is expressed in granule cells and contained in perforant path fibers (see above), but also Dyn and Enk, which may stimulate MOPr with considerable potency. MOPr receptors were detected by autoradiography in all sub-regions of the hippocampus (Slamberova et al., 2003). These MOPr are localized perisomatically, dendritically, and presynaptically on different classes of GABAergic interneurons (Drake and Milner, 2002). Activation of MOPr, like the activation of all other opioid receptors, causes a reduction of Ca^2+^ currents through P/Q-, N- and L-type channels and activation of K_ir3_ K^+^ channels through direct interaction of the βγ subunits of the G-protein with the channels (Al-Hasani and Bruchas, 2011). Moreover, MOPr couple to Gα_i_, inhibiting cAMP formation upon activation. Activation of MOPr was shown to alter synaptic plasticity in CA1, and thereby, impair spatial memory (Mansouri et al., 1997, 1999; Pourmotabbed et al., 1998). Moreover, the activation of MOPr disrupts synchronization of CA1 neuronal activity (Faulkner et al., 1998).

At present it is difficult to judge the role of MOPr in the hippocampus. Further studies on interaction with the other opioid systems are required to understand their functional role.

## The endogenous opioid system in the hippocampus: alterations in epilepsy

mTLE is associated with a number of functional, morphological, and neuropathological alterations, which impact upon the endogenous opioid system. The resulting alterations in the opioid system may contribute to or counteract seizure susceptibility, by modulation of glutamatergic and GABAergic transmission (Table 1).

**Table 1:** Alterations of the hippocampal endogenous opioid system in epilepsy.

**Alteration**	**Model**	**References**
Strong Dyn release at seizure onset, followed by Dyn depletion	Rodent kainic acid model	Kanamatsu et al., 1986b; Gall, 1988; Douglass et al., 1991; Lason et al., 1992b
Dyn depletion after seizures	Electroconvulsive shocks in rodents	Kanamatsu et al., 1986a; Xie et al., 1989b
Variable transient increase in Dyn mRNA expression after seizures	Various models	Xie et al., 1989b; Douglass et al., 1991; Lason et al., 1992a,b; Hong et al., 1993; Schwarzer and Sperk, 1998
Reduction in Dyn protein and mRNA levels	Rodent kindling models	Iadarola et al., 1986; McGinty et al., 1986; Morris et al., 1987; Lee et al., 1989; Xie et al., 1989a; Rosen et al., 1992; Harrison et al., 1995; Rocha et al., 1997
Reduced KOPr binding in CA1, reduced Dyn immunoreactivity, elevated Dyn mRNA levels	Hippocampal tissue of mesial temporal lobe epilepsy patients	de Lanerolle et al., 1997; Pirker et al., 2009
Strong release of Enk and Dyn after status epilepticus, followed by reduction of peptide levels	Rodent kainic acid model	Rocha and Maidment, 2003
Upregulated Enk expression in granule cells subsequent to seizures	Electroconvulsive shocks and kainic acid model in rodents	Hong et al., 1980; Yoshikawa et al., 1985
MOPr and DOPr change distribution patterns and function in accordance with morphological and pathological alterations	Pilocarpine and kainic acid model in rodents	Bausch and Chavkin, 1997; Skyers et al., 2003
Increased MOPr binding upon seizures	PET studies in human mTLE patients	Frost et al., 1988; Rocha et al., 2009
Brain-region specific upregulation of opioid receptor availability	PET studies in human mTLE patients	Hammers et al., 2007

### The dynorphin/KOPr system

Dyn is expressed in large quantities in mossy fibers of rodents (McGinty et al., 1983) and humans (Houser et al., 1990; Houser, 1992). This pool of Dyn is depleted during seizures due to the long-lasting, high frequency stimulation, inducing the release of large dense core vesicles. This was observed in animal models of temporal lobe epilepsy. Thus, kainic acid injection reduced Dyn levels for several hours when injected intrastriatally (Kanamatsu et al., 1986b), or several days, when given systemically to rodents (Gall, 1988; Douglass et al., 1991; Lason et al., 1992b). Electroconvulsive shocks depleted the Dyn pool for about 6 h when applied once, but up to 2 weeks upon repetitive treatment (Kanamatsu et al., 1986a; Xie et al., 1989b). This is paralleled by a transient increase in mRNA expression, ranging from 200 to 1,300% in distinct models (Xie et al., 1989b; Douglass et al., 1991; Lason et al., 1992a,b; Hong et al., 1993; Schwarzer and Sperk, 1998). This causes a transient recovery of the depleted Dyn pools. Nevertheless, Dyn levels appear subsequently decreased for a period of at least 28 days (Rocha and Maidment, 2003).

Similar reductions were reported from several kindling models of epileptogenesis (Iadarola et al., 1986; McGinty et al., 1986; Morris et al., 1987; Lee et al., 1989; Xie et al., 1989a; Rosen et al., 1992; Harrison et al., 1995). Functionally important data came from a microdialysis study, reporting significantly-reduced extracellular opioid peptide levels during the interictal period in fully kindled rats (Rocha et al., 1997). In surgically removed hippocampal tissue of mesial temporal lobe epilepsy patients, dynorphin immunoreactivity is also reduced (de Lanerolle et al., 1997), despite elevated mRNA levels in the granule cells of the hippocampus, if the patient experienced seizures within 48 h before surgery (Pirker et al., 2009).

Ca^2+^ may play a dual role in the complex regulation of expression of pDyn. Ca^2+^ activates CREB. CREB bound to CRE sites increases the activity of the pDyn promoter. On the other hand, Ca^2+^ also enhances the expression of DREAM (downstream regulatory element antagonizing modulator), which counteracts CREB by binding to the DRE (downstream regulatory element) sequence in the promoter (Cheng et al., 2002). Pronounced seizure-induced DREAM expression was shown in the mouse hippocampus (Matsu-ura et al., 2002).

Besides the regulation of pDyn expression, the Dyn/KOPr system in the hippocampus is also affected by pathological and morphological changes. Partial loss of KOPr expressing somatostatin-immunoreactive interneurons (Racz and Halasy, 2002), and pyramidal neurons are characteristic features of temporal lobe epilepsy. By contrast, mossy fibers sprout to the supergranular layer (for review see (Ben-Ari, 2001) and innervate the basal dendrites of granule cells.

Dyn immunoreactivity in tissue of patients suffering from mesial temporal lobe epilepsy differs between epilepsies with or without mossy fiber sprouting (Houser et al., 1990; de Lanerolle et al., 1997, 2003). pDyn mRNA (de Lanerolle et al., 1992) and peptide (Gall, 1988; de Lanerolle et al., 1997) were observed in hilar interneurons and CA3 pyramidal neurons in mesial temporal lobe epilepsy. This was neither observed in healthy brain nor in epilepsies without hippocampal sclerosis and mossy fiber sprouting, such as mass-associated or paradoxical temporal lobe epilepsy (Hurd, 1996). Reduced tissue levels of Dyn-immunoreactivity in mTLE (de Lanerolle et al., 1997) may be due to neuronal loss, excessive release during seizures (Sperk et al., 1986; Marksteiner et al., 1989; McDermott and Schrader, 2011), or Dyn down-regulation.

Like for Dyn–immunoreactivity, the hippocampi of patients suffering from mass-associated or paradoxical temporal lobe epilepsy displayed similar [^3^H]U69,593 binding as post-mortem controls. Reduced binding in area CA1 in mTLE patients appears to be dependent on neuronal loss, as the subiculum is spared from both (de Lanerolle et al., 1997).

The loss of Dyn, probably resulting in a lack of inhibition of voltage-gated Ca^2+^ currents in the hippocampal granule cells (Jeub et al., 1999), may be functionally important. Increased Ca^2+^ currents lead to augmented glutamate release, thereby facilitating the generation of seizures. Of note is the fact, that the loss of inhibition of voltage-gated Ca^2+^ currents was closely associated with mossy fiber sprouting and hippocampal sclerosis.

Overall, alterations in the Dyn/KOPr system in epilepsy suggests a loss of inhibition on glutamatergic neurons (Table 1). This may contribute to the progression of disease development and severity. However, the depletion of Dyn, whilst conservation of the KOPr offers the possibility of pharmacological intervention.

### The enkephalin/DOPr system and MOPr

The expression of Enk in the hippocampi of naive rodents is mostly restricted to interneurons. However, Enk expression is upregulated in granule cells subsequent to seizures (Hong et al., 1980; Yoshikawa et al., 1985). In the model of unilateral injection of kainic acid into the dorsal hippocampus of mice, the upregulation of pEnk mRNA appeared to be independent of seizures, which is distinct of the regulation of other neuropeptides like pDyn or pNPY. The promoter of the Enk gene contains 2 CRE sites, that regulate the cAMP and phorbol-ester-inducible expression of pEnk in conjuction with a downstream AP-2 site (Comb et al., 1986, 1988; Grove et al., 1989). Moreover, PKA was shown to influence the activity of the human pEnk promoter (Huggenvik et al., 1991). In human patients, β-endorphin appears to be elevated in the CSF postictally (Pitkanen et al., 1987). Judging from the plasma levels of the patients, Marek et al. (2010) suggest that β-endorphin concentrations are related to the frequency of seizures and the duration of the disease, while leu-enkephalin concentrations are related primarily to the duration of the disease.

Under physiological conditions, DOPr and MOPr are distributed in diffuse patterns in the hippocampus, as shown by receptor autoradiography (Mansour et al., 1988), with MOPr being more prominent, however, they are relatively distant from synapses (Drake and Milner, 1999). Upon seizures, both MOPr and DOPr have been reported to change their distribution patterns and function in accordance with both morphological and pathological alterations (Bausch and Chavkin, 1997; Skyers et al., 2003). The number of DOPr or MOPr immunopositive neurons in the hippocampus appears to be reduced in both, the hilus and granule cell layer. By contrast, diffuse immunoreactivity for DOPr and MOPr appeared increased in the inner molecular layer in the pilocarpine model of TLE (Bausch and Chavkin, 1997). The increase in MOPr in the inner molecular layer may be associated with a variety of fibers originating from granule cells or surviving GABAergic interneurons, as well as septal or supramamillary projections (Skyers et al., 2003). Receptor binding of MOPr also increases upon seizures in human mTLE, however, this effect seems to be restricted mainly to the temporal cortex (Frost et al., 1988; Rocha et al., 2009). Specific MOPr splice variants were observed in some forms of intractable epilepsy (Fricchione et al., 2008), however, their function remains unclear.

In epilepsy numerous GABAergic interneurons die, suggesting a large reduction of neurons expressing DOPr or MOPr. However, survival of interneurons in animal models of TLE differ quite significantly from human conditions, thus translatability of rodent studies has to be considered carefully (Table 1).

## The endogenous opioid system in the hippocampus: implications in epilepsy

Opioids, and, in particular, dynorphin, have been implicated in the modulation of neuronal excitability *in-vitro* (Henriksen et al., 1982; Siggins et al., 1986). *In-vivo*, opioid receptors, with their ligands form neuromodulatory systems, playing major roles not only in nociceptive pathways, in affective behavior, neuroendocrine physiology, and autonomic functions (Kieffer and Evans, 2009), but also in epilepsy, and importantly, through their action in the hippocampus. That the opioid systems modulate seizure activity has been shown by opiates (Hong et al., 1993), and that opioid receptors adapt following spontaneous seizures has been demonstrated by using a non-subtype selective opioid receptor PET radioligand, showing a brain-region specific upregulation of opioid receptor availability (Hammers et al., 2007).

**Figure 1:**
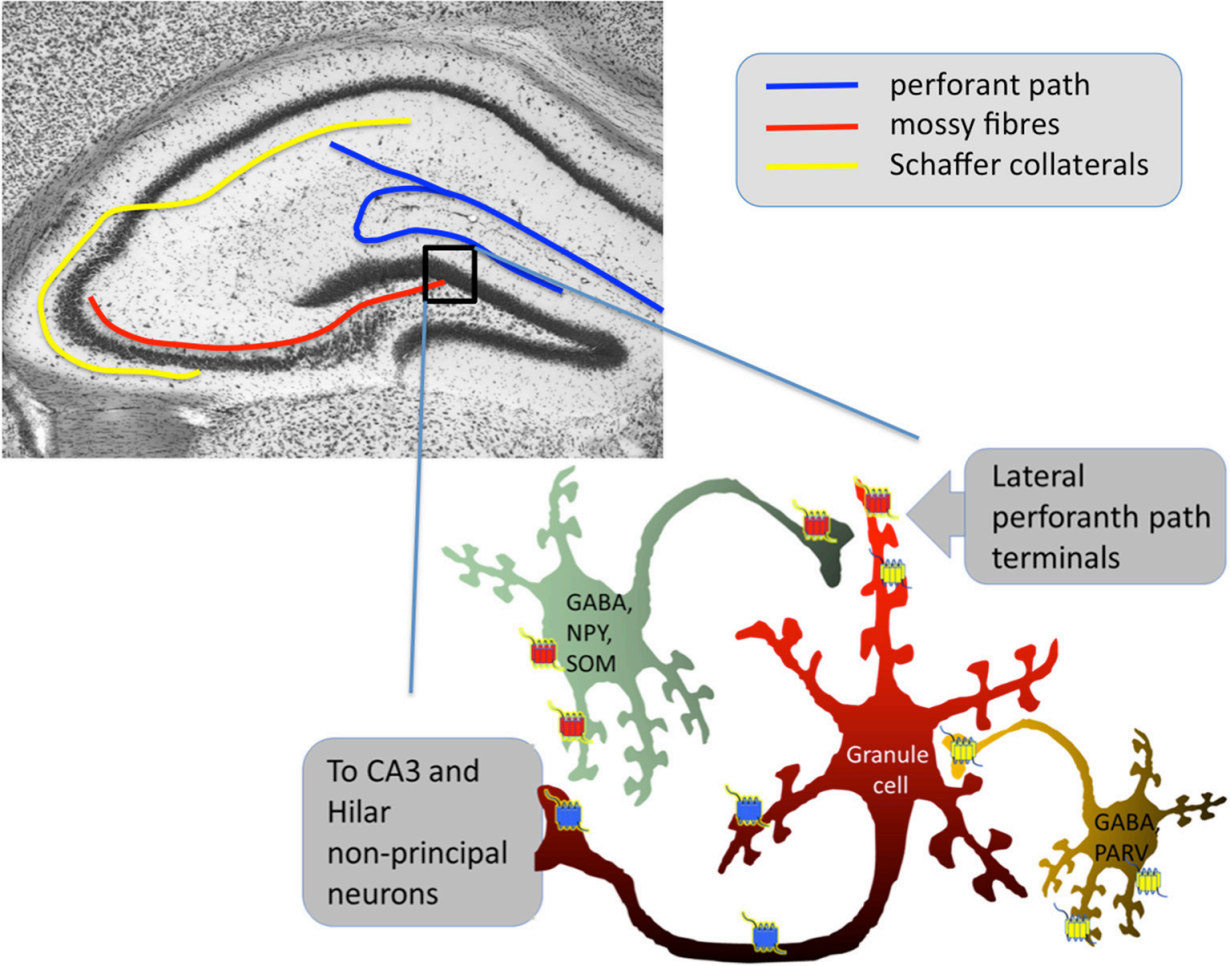
Simplified scheme of hippocampal dentate gyrus network control by opioid receptors. Blue represents KOPr, red DOPr and yellow MOPr for more detailed descriptions of the individual opioid systems' functions in the hippocampus, see Drake and Milner (1999), Rezai et al. (2012), and Schwarzer (2009). DOPr are often located on GABAergic neurons containing neuropeptide Y (NPY) and somatostatin (SOM) (Commons and Milner, 1996), MOPr are often on GABAergic neurons containing parvalbumin (PARV) (Drake and Milner, 1999). Note that especially DOPr and MOPr could also be active as heterodimers. Activation of both, MOPr and DOPr has predominantly disinhibitory effects on granule cells.

Dyn and Enk, despite the similar molecular actions of their preferential receptors (KOPr and DOPr/MOPr) exert very different effects on seizure-induction, most likely due to their differential localization within the hippocampus (see Figure 1). However, though KOPr is predominantly expressed on glutamatergic neurons and their activation yields a net inhibitory effect, DOPr and MOPr are often located on neurons inhibiting glutamatergic principal neurons, thus, their activation may results in net disinhibitory effects (Table 2).

**Table 2:** Implications of the hippocampal endogenous opioid system on excitability, epilepsy, and neuroprotective potentials.

**Implications**	**Model**	**References**
Stimulation of hippocampal granule cells inhibits perforant path terminals potentially via presynaptic KOPr on perforant path axons	Guinea pig, rodents	Wagner et al., 1993; Drake et al., 1994; Salin et al., 1995
Stimulation of mossy fibers inhibits neighboring mossy fibers via Dyn	Guinea pig	Weisskopf et al., 1993
Dyn exerts anticonvulsant effects	Various rodent models	Tortella et al., 1986, 1989, 1990; Przewlocka et al., 1995; Solbrig et al., 2006; Loacker et al., 2007
Antiepileptogenic effects of Dyn is mediated via the KOPr	Rodent kainic acid model	Loacker et al., 2007
Low pDyn levels due to mutations in the promoter regions result in increased vulnerability for epilepsy	Human epilepsy patients	Stogmann et al., 2002; Gambardella et al., 2003
KOPr activation in periods of low Dyn suppresses seizures	Various rodent models	Tortella, 1988; Takahashi et al., 1990; Solbrig et al., 2006; Loacker et al., 2007
KOPr activation during epileptogenesis increases neuronal survival	Rodent kainic acid model	Schunk et al., 2011
Pharmacological MOPr or DOPr activation has disinhibititory effects and facilitats synaptic plasticity/seizure susceptibility	Rodents	Neumaier et al., 1988; Cohen et al., 1992; Lupica, 1995
DOPr inhibition prevents, and DOPr-activation facilitates self-sustained status epilepticus	Performant path stimulation in rats	Mazarati et al., 1999
Subcutaneous DOPr agonist administration produce convulsions	Rodent kindling model	Broom et al., 2002; Jutkiewicz et al., 2006
DOPr agonists produce moderate convulsions	Non-human primate kindling model	Negus et al., 1998; Danielsson et al., 2006
Intrahippocampal β-endorphin injections result in generalized convulsions, administration into the ventricle strongly reduces this effect	Rodent kindling model	Cain et al., 1990
Neuroprotection of DOPr activation	Hypoxia/ischemia, glutamate-induced excitotoxic injury and oxidative stress models	Mayfield and D'Alecy, 1994; Zhang et al., 2000; Narita et al., 2006; Yang et al., 2009

### The dynorphin/KOPr system

The dominant effect of the endogenous dynorphin is anticonvulsant (Tortella et al., 1986, 1989, 1990; Przewlocka et al., 1995; Solbrig et al., 2006), antiepileptogenic, and is mediated via the kappa opioid receptor (Loacker et al., 2007). Deletion of the coding region of the prodynorphin gene in mice resulted in an increased seizure susceptibility and affected neurodegeneration during epileptogenesis. In line with this, low prodynorphin levels, due to mutations in the promoter regions in humans (Stogmann et al., 2002; Gambardella et al., 2003), result in an increased vulnerability toward epilepsy.

### The enkephalin/DOPr system and MOPr

Mazarati et al. (1999) showed that DOPr inhibition prevents, and DOPr-activation facilitates self-sustained status epilepticus in a model of perforant path stimulation. These effects are, however, strongly dependent on the applied agonists (Saitoh et al., 2011; Clynen et al., 2014; Chung et al., 2015), and might differ across species. For example, the DOPr agonist SNC80 appears to produce stronger convulsions in the rat (Broom et al., 2002; Jutkiewicz et al., 2006), than in the rhesus monkey (Negus et al., 1998; Danielsson et al., 2006). Induction of seizures, furthermore, might be predominantly depending on the DOPr activation on forebrain GABAergic neurons, as Chung et al. (2015) demonstrated using SNC80 on mice with DOPr knocked out specifically in those neurons.

Due to the mainly inhibitory net effects of MOPr in the hippocampus, microinjections of β-endorphin into the hippocampus result in generalized convulsions, however, administration into the ventricle strongly reduces this effect (Cain et al., 1990). Systemic administration of MOPr agonists occasionally even result in anti-convulsant effects, maybe due to the differential actions in other brain regions (reviewed in Simmons and Chavkin, 1996). Still, seizure development appears to be more dependent on MOPr activation than on DOPr activation, as the former, but not the latter, causes convulsions (Lee et al., 1989; Hong et al., 1993).

Interestingly, agonists at DOPr can block certain effects of MOPr agonists and vice versa (ONeill et al., 1997), potentially reflecting the competition of the different agonists at the receptor subtypes or heterodimerization.

## The endogenous opioid system in the hippocampus: potentials in epilepsy therapy

The patterns of regulations during epileptogenesis differ strongly between Dyn and Enk, which is demonstrated for mRNAs in response to status epilepticus (Hong et al., 1993). While the increase of pDyn mRNA was transient, followed by a reduction, pEnk expression appeared to be lastingly increased. Whether such continuous Enk mRNA upregulation plays a role in promoting epileptogenesis or in counteracting effects requires further investigation, and that has been discussed below (Table 2). With respect to the described anti-convulsant properties of Dyn, reduced mRNA levels suggest that the application of exogenous KOPr-agonists in these periods may be beneficial.

### The dynorphin/KOPr system

The approach to activate KOPr in the periods of low Dyn has indeed been shown to have a potential to suppress seizures (Tortella, 1988; Takahashi et al., 1990; Solbrig et al., 2006; Loacker et al., 2007), and it increases the survival of neurons in the hippocampus and amygdala after unilateral injection of kainic acid into the hippocampus of mice (Schunk et al., 2011). Clinical trials, using the full KOPr agonists spiradoline or enadoline, have failed due to dysphoric side-effects in the 1990s (Barber and Gottschlich, 1997; Schwarzer, 2009). As a consequence, industrial research has been essentially discontinued. Recently, we reported that by using biased KOPr-agonists, the anticonvulsant/antiseizure effects can be separated from the dysphoric effects (Zangrandi et al., 2016), opening new therapeutical potentials.

A promising approach to target the opioid system and counteract seizures in a disease modifying way, is adeno-associated virus (AAV) gene therapy. AAV-gene therapy is a promising tool to target a broad array of neurological diseases (Weinberg et al., 2013). Several pre-clinical studies on AAV-mediated gene-delivery of neuropeptides are already available (for a review see Kovac and Walker, 2013). Gene-therapy for Dyn might be an interesting approach to activate KOPr and achieve anti-convulsant effects through replenishing Dyn in different phases of depletion of endogenous Dyn. Due to local restriction of the therapy, side effects known from systemic application of KOPr agonists may be avoided.

### The enkephalin/DOPr system

The described pro-convulsant properties of some DOPr agonists and the initial continuous upregulation of Enk mRNA during epileptogensis suggest Enk to act as a potential driving force of epileptogenesis. Interestingly, however, the activation of DOPr has also been implicated in neuroprotection, suggesting their potential dual role in epilepsy; upregulation during epileptogenesis might be beneficial, even though the net effect of DOPr activation seems to be pro-convulsant. Neuroprotection of DOPr has been reported specifically for hypoxia/ischemia (Mayfield and D'Alecy, 1994; Zhang et al., 2000; Yang et al., 2009), glutamate-induced excitotoxic injury (Zhang et al., 2000), and oxidative stress (Narita et al., 2006), potentially via their positive effects on mitochondrial function (Zhu et al., 2009, 2011). Leu-Enk peptide has been shown to be upregulated in hippocampus and cortex after hypoxic preconditioning (Gao et al., 2012). Also, DOPr is upregulated through hypoxic preconditioning, and a decrease of Leu-Enk during severe hypoxia is inhibited, counteracting the increased p38 MAPK activity, cytochrome c release and apoptosis, induced by severe hypoxia (Ma et al., 2005).

Furthermore, the differential effects of different MOPr agonists, the complexity and dynamics of the Enk/MOPr system, and the dubiety whether the hippocampal Enk/MOPr system is required for MOPr induced seizures warrant further elucidation of this system's involvement, especially in epileptogenesis.

Importantly, the convulsive properties of some DOPr agonists represent a major drawback in their great pharmacological potentials for chronic pain (reviewed by Gaveriaux-Ruff and Kieffer, 2011) and mood disorders (reviewed by Chung and Kieffer, 2013); DOPr are importantly involved in the control of emotional responses, such as anxiety and depression-like behaviors (Filliol et al., 2000; Pradhan et al., 2011). An anxiogenic phenotype has been reported for both, DOPr- (Filliol et al., 2000) and Enkephalin-knockout (Konig et al., 1996) mice, which is not the case in mice deficient for KOPr or MOPr. Accordingly, pharmacological tools have been used to target many of these behavioral effects of the DOPr/Enk system (Pradhan et al., 2011). In general, DOPr agonists tested in clinical trials for other applications (like ADL5859 and 5747, AZD5258 and AZD2327), appeared to have elicited no severe adverse effects, and were, overall, well-tolerated (Charles and Pradhan, 2016; Richards et al., 2016). Therefore, DOPr agonists (without proconvulsive potential) represent promising drug-candidates, despite all of the mentioned examples failing to reach their primary clinical endpoints for their respective purposes.

The availability of MOPr in the hippocampus and its involvement in seizure susceptibility makes it a potential target in epilepsy and seizures. This system is understudied in epilepsy research, however, owing probably also to a lack of primary endogenous ligands to study regulations in epilepsy.

## Conclusion

In conclusion, a better understanding of the complex opioid system in the hippocampus, including functionally selective agonists and di-/oligomerizations of opioid receptors and neuroprotective effects (in particular of DOPr), is giving rise to new therapeutic concepts, and will drive research on new medical applications of the opioid systems. Specifically, the Dyn/KOPr system bears great potentials to target epilepsy and epileptogenesis, also for the fashioning of disease-modifying treatments, with the possibility of the elimination of side-effects through design, and the selection of relevant agonists and attractive delivery methods, such as gene therapy approaches. Pro-convulsant properties of DOPr activation can be avoided by the selection of adequate agonists with the desired functional selectivity to specifically exploit the neuroprotective potential of DOPr activation. Yet, the neuroprotective effects of DOPr activation require further investigations. Providing the identification of DORr agonists, which conserve the neuroprotective but lack proconvulsant effects, may be of great interest in the course of epilepsy therapy.

## Author contributions

All authors listed, have made substantial, direct and intellectual contribution to the work, and approved it for publication.

### Conflict of interest statement

CS has a patent application pending for the use of preprodynorphin viral vectors in gene therapy for focal epilepsy. The other author declares that the research was conducted in the absence of any commercial or financial relationships that could be construed as a potential conflict of interest.
